# Supplementary material for: Human Melanocortin-2 Receptor: Identifying a Role for Residues in the TM4, EC2, and TM5 Domains in Activation and Trafficking as a Result of Co-Expression with the Accessory Protein, Mrap1 in Chinese Hamster Ovary Cells
Source: Biomolecules. 2022 Oct 4;12(10):1422. doi: 10.3390/biom12101422 (PMC9599514; doi:10.3390/biom12101422)
Supplement: Supplementary file 1 [file biomolecules-12-01422-s001.zip › Supplementary Figures and Tables/Supplementary Table S1 Nucleotide Sequences of hMC2R xtMc1r Chimeric receptors.pdf]

Supplementary Table S1: Nucleotide Sequences

Human MC2R (accession number: AA067714.1)

ATGAAGCACATTATCAACTCGTATGAAAACATCAACAACACAGCAAGAAATAATTCCGACTGTCCTCGTGTGGTTTT
GCCGGAGGAGATATTTTTCACAATTTCCATTGTTGGAGTTTTGGAGAATCTGATCGTCCTGCTGGCTGTGTTCAAGA
ATAAGAATCTCCAGGCACCCATGTACTTTTTCATCTGTAGCTTGGCCATATCTGATATGCTGGGCAGCCTATATAAG
ATCTTGGAAAATATCCTGATCATATTGAGAAACATGGGCTATCTCAAGCCACGTGGCAGTTTTGAAACCACAGCCGA
TGACATCATCGACTCCCTGTTTGTCCTCTCCCTGCTTGGCTCCATCTTCAGCCTGTCTGTGATTGCTGCGGACCGCT
ACATCACCATCTTCCACGCACTGCGGTACCACAGCATCGTGACCATGCGCCGCACTGTGGTGGTGCTTACGGTCATC
TGGACGTTCTGCACGGGGACTGGCATCACCATGGTGATCTTCTCCCATCATGTGCCCACAGTGATCACCTTCACGTC
GCTGTTCCCGCTGATGCTGGTCTTCATCCTGTGCCTCTATGTGCACATGTTCCTGCTGGCTCGATCCCACACCAGGA
AGATCTCCACCCTCCCCAGAGCCAACATGAAAGGGGCCATCACACTGACCATCCTGCTCGGGGTCTTCATCTTCTGC
TGGGCCCCCTTTGTGCTTCATGTCCTCTTGATGACATTCTGCCCAAGTAACCCCTACTGCGCCTGCTACATGTCTCT
CTTCCAGGTGAACGGCATGTTGATCATGTGCAATGCCGTCATTGACCCCTTCATATATGCCTTCCGGAGCCCAGAGC
TCAGGGACGCATTCAAAAAGATGATCTTCTGCAGCAGGTACTGGTGA

Xenopus tropicalis Mc1r (accession number: XP 012817790)

ATGCTTCACTCAACAGTCAACTCCACCAATGCCACCATCAATGTTGGGACTGAACTTAAGCCAACCAACACATCCGA
CACTGTTATGGACGTTCCAGAAGAGCTCTTTCTGTTCCTGTGTGTGTTCAGTTTGCTAGAAAACATACTTGTGGTCA
TTGCCATTTTTAGGAACCACAACCTGCACTCTCCCATGTATTATTTCATCTGCTGCCTGGCTGCATCTGATATGCTT
GTAAGTTCTAGCAACCTAGGTGAGACCCTCATCATTTTTATGCTTAAGCAAGGGATTATAAAATCTGAGCCACTGCT
TGTCAAAAAAATGGATTATATATTTGATACAATGATCTGCTGCTCTTTAGTAACCTCTCTCTCCTTTCTTGGGGCCA
TTGCAATTGACCGATACATCACCATCTTCTATGCCTTGCGCTATCACAGCATTATGACACTGCGCAGAGTAGTTATT
GCCATAGGCGTGATCTGGTCGGTCAGTTTAGTTTGTGCGGCCATATTCATTGTCTATCATGAGAGTCGTGCTGTCAT
CTTATGTCTTATTGTATTTTTTCTCTTCATGTTGGCCTTGATGGTGGCTTTATATATCCACATGTTTGCTCTGGCTC
GCCAGCACGCACGTAGCATCTCTGCCCTGCAGAAGGGGAAGAGTAGGAGAATTACCCCACATCAAGCTAGGGCAAAC
ATGAAAGGAGCCATAACACTAACATTGTTGCTTGGCGTCTTTTTTTCTTTGTTGGGGGCCTCTGTTCCTCCACCTCAC
TTTGTTTGTGTCCTGCCCCGGTCATCATATTTGCAATAGCTATTTTTACTATTTCAACATCTACCTCCTTCTTGTCA
TCTGCAATTCTGTTATTGACCCCTTAATCTACGCCTTCAGGAGTCAGGAGCTAAGGAAGACCCTCAAGGAAATTGTC
TGGTGCTCTTGGTGA

Chimeric hMC2R/N-terminal xtMC1R (in red) Cys codon underlined

ATGCTTCACTCAACAGTCAACTCCACCAATGCCACCATCAATGTTGGGACTGAACTTAAGCCAACCAACACATCCTG
CACTGTTATGGACGTTCCAGAAGAGATATTTTTCACAATTTCCATTGTTGGAGTTTTGGAGAATCTGATCGTCCTGC
TGGCTGTGTTCAAGAATAAGAATCTCCAGGCACCCATGTACTTTTTCATCTGTAGCTTGGCCATATCTGATATGCTG
GGCAGCCTATATAAGATCTTGGAAAATATCCTGATCATATTGAGAAACATGGGCTATCTCAAGCCACGTGGCAGTTT
TGAAACCACAGCCGATGACATCATCGACTCCCTGTTTGTCCTCTCCCTGCTTGGCTCCATCTTCAGCCTGTCTGTGA
TTGCTGCGGACCGCTACATCACCATCTTCCACGCACTGCGGTACCACAGCATCGTGACCATGCGCCGCACTGTGGTG
GTGCTTACGGTCATCTGGACGTTCTGCACGGGGACTGGCATCACCATGGTGATCTTCTCCCATCATGTGCCCACAGT
GATCACCTTCACGTCGCTGTTCCCGCTGATGCTGGTCTTCATCCTGTGCCTCTATGTGCACATGTTCCTGCTGGCTC
GATCCCACACCAGGAAGATCTCCACCCTCCCCAGAGCCAACATGAAAGGGGCCATCACACTGACCATCCTGCTCGGG
GTCTTCATCTTCTGCTGGGCCCCCTTTGTGCTTCATGTCCTCTTGATGACATTCTGCCCAAGTAACCCCTACTGCGC
CTGCTACATGTCTCTCTTCCAGGTGAACGGCATGTTGATCATGTGCAATGCCGTCATTGACCCCTTCATATATGCCT
TCCGGAGCCCAGAGCTCAGGGACGCATTCAAAAAGATGATCTTCTGCAGCAGGTACTGGTAG

Chimeric hMC2R/EC1 xtMC1R (in red)

ATGAAGCACATTATCAACTCGTATGAAAACATCAACAACACAGCAAGAAATAATTCCGACTGTCCTCGTGTGGTTTT
GCCGGAGGAGATATTTTTCACAATTTCCATTGTTGGAGTTTTGGAGAATCTGATCGTCCTGCTGGCTGTGTTCAAGA
ATAAGAATCTCCAGGCACCCATGTACTTTTTCATCTGTAGCTTGGCCATATCTGATATGCTGGGCAGCCTATATAAG
ATCTTGGAAAATATCCTGATTTTTATGCTTAAGCAAGGGATTATAAAATCTGAGCCACTGCTTGTCAAAAAAATGGA
TGACATCATCGACTCCCTGTTTGTCCTCTCCCTGCTTGGCTCCATCTTCAGCCTGTCTGTGATTGCTGCGGACCGCT
ACATCACCATCTTCCACGCACTGCGGTACCACAGCATCGTGACCATGCGCCGCACTGTGGTGGTGCTTACGGTCATC
TGGACGTTCTGCACGGGGACTGGCATCACCATGGTGATCTTCTCCCATCATGTGCCCACAGTGATCACCTTCACGTC
GCTGTTCCCGCTGATGCTGGTCTTCATCCTGTGCCTCTATGTGCACATGTTCCTGCTGGCTCGATCCCACACCAGGA
AGATCTCCACCCTCCCCAGAGCCAACATGAAAGGGGCCATCACACTGACCATCCTGCTCGGGGTCTTCATCTTCTGC
TGGGCCCCCTTTGTGCTTCATGTCCTCTTGATGACATTCTGCCCAAGTAACCCCTACTGCGCCTGCTACATGTCTCT
CTTCCAGGTGAACGGCATGTTGATCATGTGCAATGCCGTCATTGACCCCTTCATATATGCCTTCCGGAGCCCAGAGC
TCAGGGACGCATTCAAAAAGATGATCTTCTGCAGCAGGTACTGGTGA

Chimeric hMC2R/EC2 xtMC1R (in red)

ATGAAGCACATTATCAACTCGTATGAAAACATCAACAACACAGCAAGAAATAATTCCGACTGTCCTCGTGTGGTTTT
GCCGGAGGAGATATTTTTCACAATTTCCATTGTTGGAGTTTTGGAGAATCTGATCGTCCTGCTGGCTGTGTTCAAGA
ATAAGAATCTCCAGGCACCCATGTACTTTTTCATCTGTAGCTTGGCCATATCTGATATGCTGGGCAGCCTATATAAG
ATCTTGGAAAATATCCTGATCATATTGAGAAACATGGGCTATCTCAAGCCACGTGGCAGTTTTGAAACCACAGCCGA
TGACATCATCGACTCCCTGTTTGTCCTCTCCCTGCTTGGCTCCATCTTCAGCCTGTCTGTGATTGCTGCGGACCGCT
ACATCACCATCTTCCACGCACTGCGGTACCACAGCATCGTGACCATGCGCCGCACTGTGGTGGTGCTTACGGTCATC
TGGACGTTCTGCACGGGGACTGGCATCACCATGGTGATCTATCATGAGAGTCGTGCTGTCATCTTAACCTTCACGTC
GCTGTTCCCGCTGATGCTGGTCTTCATCCTGTGCCTCTATGTGCACATGTTCCTGCTGGCTCGATCCCACACCAGGA
AGATCTCCACCCTCCCCAGAGCCAACATGAAAGGGGCCATCACACTGACCATCCTGCTCGGGGTCTTCATCTTCTGC
TGGGCCCCCTTTGTGCTTCATGTCCTCTTGATGACATTCTGCCCAAGTAACCCCTACTGCGCCTGCTACATGTCTCT
CTTCCAGGTGAACGGCATGTTGATCATGTGCAATGCCGTCATTGACCCCTTCATATATGCCTTCCGGAGCCCAGAGC
TCAGGGACGCATTCAAAAAGATGATCTTCTGCAGCAGGTACTGGTGA

Chimeric hMC2R/EC3 xtMc1r (in red) Cys codon underlined

ATGAAGCACATTATCAACTCGTATGAAAACATCAACAACACAGCAAGAAATAATTCCGACTGTCCTCGTGTGGTTTT
GCCGGAGGAGATATTTTTCACAATTTCCATTGTTGGAGTTTTGGAGAATCTGATCGTCCTGCTGGCTGTGTTCAAGA
ATAAGAATCTCCAGGCACCCATGTACTTTTTCATCTGTAGCTTGGCCATATCTGATATGCTGGGCAGCCTATATAAG
ATCTTGGAAAATATCCTGATCATATTGAGAAACATGGGCTATCTCAAGCCACGTGGCAGTTTTGAAACCACAGCCGA
TGACATCATCGACTCCCTGTTTGTCCTCTCCCTGCTTGGCTCCATCTTCAGCCTGTCTGTGATTGCTGCGGACCGCT
ACATCACCATCTTCCACGCACTGCGGTACCACAGCATCGTGACCATGCGCCGCACTGTGGTGGTGCTTACGGTCATC
TGGACGTTCTGCACGGGGACTGGCATCACCATGGTGATCTTCTCCCATCATGTGCCCACAGTGATCACCTTCACGTC
GCTGTTCCCGCTGATGCTGGTCTTCATCCTGTGCCTCTATGTGCACATGTTCCTGCTGGCTCGATCCCACACCAGGA
AGATCTCCACCCTCCCCAGAGCCnAACATGAAAGGGGCCATCACACTGACCATCCTGCTCGGGGTCTTCATCTTCTG
CTGGGCCCCCTTTGTGCTTCATGTCCTCTTGATGGTGTTCTGCCCCGGTCATCATATTTGCAATTGCTATTTTTACT
ATTTCCAGGTGAACGGCATGTTGATCATGTGCAATGCCGTCATTGACCCCTTCATATATGCCTTCCGGAGCCCAGAG
CTCAGGGACGCATTCAAAAAGATGATCTTCTGCAGCAGGTACTGGTGA

The nucleotide sequences for *hmc2r* and *xtmc1r* and the four chimeric receptor cDNAs: hMC2R/N-terminal xtMC1R, Chimeric hMC2R/EC1 xtMC1R, hMC2R/EC2 xtMC1R,and hMC2R/EC3 xtMc1r are presented. The location the xtMc1r nucleotide sequence in each chimeric construct is highlighted in red. The location of the cysteine codon in the NT and EC3 chimeric constructs is highlighted in green and underlined.
