# Supplementary material for: Human Melanocortin-2 Receptor: Identifying a Role for Residues in the TM4, EC2, and TM5 Domains in Activation and Trafficking as a Result of Co-Expression with the Accessory Protein, Mrap1 in Chinese Hamster Ovary Cells
Source: Biomolecules. 2022 Oct 4;12(10):1422. doi: 10.3390/biom12101422 (PMC9599514; doi:10.3390/biom12101422)
Supplement: Supplementary file 1 [file biomolecules-12-01422-s001.zip › Supplementary Figures and Tables/Supplementary Table S2.pdf]

Supplementary Table S2

Comparison of Vmax values for hMC2r/xtMc1r Chimeric Receptors

Receptor	Vmax* Luciferase Units mean+S.E.M; n=3)	Percent Change in Vmax Value Relative to hMC2R
hMC2R	128380±2439	-
hMC2R/NT xtMc1r	199367±8215	+55%
hMC2R/EC1 xtMc1r	102990±1170	-20%
hMC2R/EC2 xtMc1r	94956+11086[1]	-35%
hMC2R/EC3 xtMc1r	137840±2240	+ 7%

*Vmax was calculated using the Kaleidograph figure plotting software.

[1]extrapolated from Figure 2B using Kadeidograph software.
