# Supplementary material for: Human Melanocortin-2 Receptor: Identifying a Role for Residues in the TM4, EC2, and TM5 Domains in Activation and Trafficking as a Result of Co-Expression with the Accessory Protein, Mrap1 in Chinese Hamster Ovary Cells
Source: Biomolecules. 2022 Oct 4;12(10):1422. doi: 10.3390/biom12101422 (PMC9599514; doi:10.3390/biom12101422)
Supplement: Supplementary file 1 [file biomolecules-12-01422-s001.zip › Supplementary Figures and Tables/Supplementary Table S3.pdf]

Supplementary Table S3

Cell Surface ELIZA of Single Alanine Mutants of hMC2R

TM4 (A)	Average S.E.M (n=3)	One-Way ANOVA p Value
Negative	0.162 ± 0.001	< 0.0001
Positive	0.792 ± 0.091	-
G162	1.420 ± 0.081	< 0.0001
I163	0.380 ± 0.037	0.0016
T164	0.705 ± 0.011	0.666

TM4 (B)	Average S.E.M (n=3)	p Value
Negative	0.236 ± 0.016	< 0.0001
Positive	0.598 ± 0.091	-
M165	0.198 ± 0.016	< 0.0001
V166	0.354 ± 0.018	< 0.0001
I167	0.530 ± 0.035	0.1275

TM5 (C)	Average S.E.M (n=3)	p Value
Negative	0.125 ± 0.033	0.004
Positive	0.423 ± 0.027	-
T177	0.721 ± 0.043	0.0004
F178	0.212 ± 0.032	0.0053
T179	0.419 ± 0.037	0.9954

TM5 (D)	Average S.E.M (n=3)	p Value
Negative	0.182 ± 0.031	0.0447
Positive	0.496 ± 0.061	-
S180	1.496 ± 0.076	< 0.0001
L181	0.518 ± 0.126	0.9997
F182	0.461 ± 0.861	0.9983
P183	0.861 ± 0.100	0.443

EC2 (E)	Average S.E.M (n=3)	p Value
Negative	0.219 ± 0.003	< 0.0001
Positive	1.364 ± 0.035	-
F168	0.413 ± 0.272	0.0001
H170	1.171 ± 0.157	0.3123

Negative Control - hMC2R expressed alone in CHO cells
Positive Control - hMC2R co-expressed with mMrap1 in CHO cells
All mutants were co-expressed with mMrap1 Mutants in highlighted in red resulted in a statistically significant drop in trafficking
