# Supplementary figures and images for: Human Melanocortin-2 Receptor: Identifying a Role for Residues in the TM4, EC2, and TM5 Domains in Activation and Trafficking as a Result of Co-Expression with the Accessory Protein, Mrap1 in Chinese Hamster Ovary Cells
Source: Biomolecules. 2022 Oct 4;12(10):1422. doi: 10.3390/biom12101422 (PMC9599514; doi:10.3390/biom12101422)

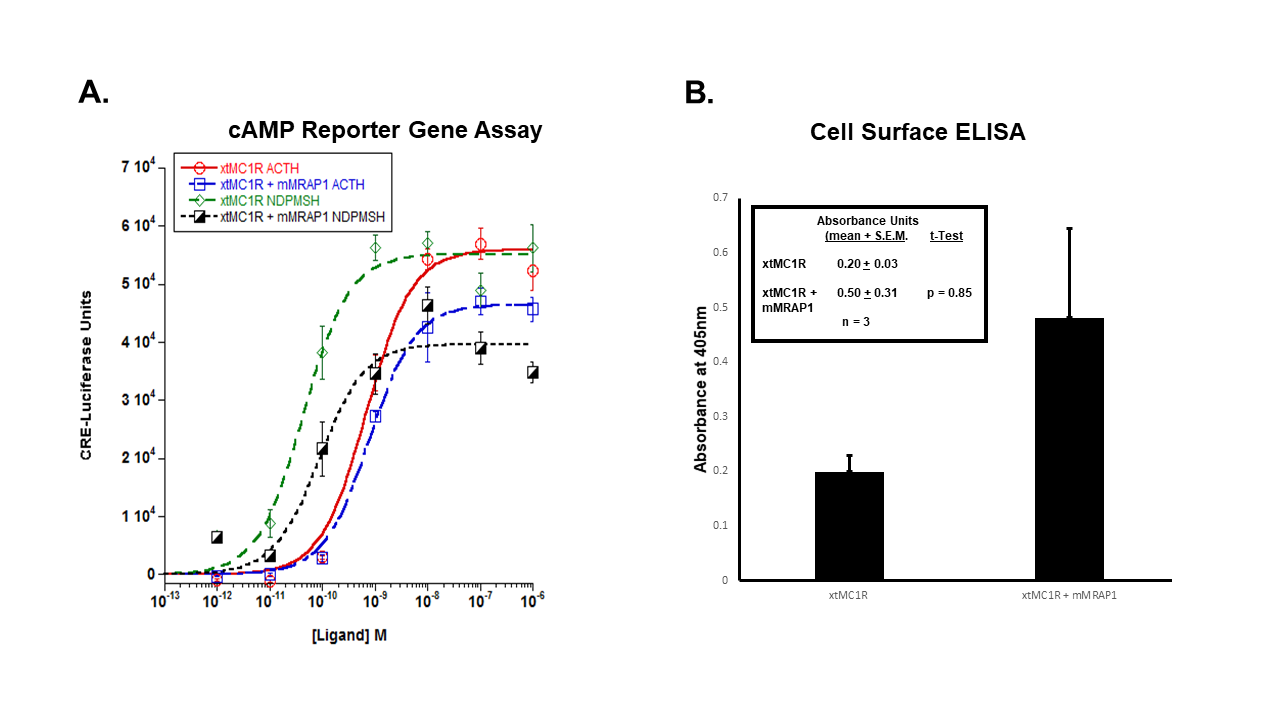

Supplement: Supplementary file 1 [file biomolecules-12-01422-s001.zip › Supplementary Figures and Tables/Figure S1.tif]
